# The Efficacy and Safety of Phase I Cardiac Rehabilitation in Patients Hospitalized in Cardiac Intensive Care Unit With Acute Decompensated Heart Failure: A Study Protocol for a Randomized, Controlled, Clinical Trial

**DOI:** 10.3389/fcvm.2022.788503

**Published:** 2022-03-08

**Authors:** Linjing Wu, Jiahua Li, Linjian Chen, Mengmeng Xue, Yamin Zheng, Fanqi Meng, Hongfei Jiang, Zaixing Shi, Peng Zhang, Cuilian Dai

**Affiliations:** ^1^Department of Cardiology, Xiamen Cardiovascular Hospital of Xiamen University, School of Medcine, Xiamen University, Xiamen, China; ^2^Key Laboratory of Health Technology Assessment of Fujian Province, School of Public Health, Xiamen University, Xiamen, China

**Keywords:** acute decompensated heart failure, hospitalization, cardiac rehabilitation, cardiac intensive care unit, short physical performance battery

## Abstract

**Background:**

Cardiac rehabilitation (CR) improves outcomes in patients with heart failure. However, data on CR efficacy in patients with acute decompensated heart failure is limited. This study is designed to assess the efficacy and safety of CR in patients hospitalized in cardiac intensive care unit (CICU) with acute decompensated heart failure (ADHF).

**Methods:**

This is a single-center, randomized controlled, single-blind clinical trial. A total of 120 participants hospitalized in CICU with ADHF will be randomly allocated in the ratio of 1:1 to two groups: CR group and control group. Participants will receive tailored and progressive CR intervention or attention control. The CR intervention include personalized breathing training, small muscle group resistance training, and aerobic endurance training based on the physical fitness assessment results. The subjects will receive the CR training for 5 days and will be followed up for 6 months. The primary endpoints are the score of the short physical performance battery (SPPB) and 6-month all-cause rehospitalization. The secondary endpoints include cardio-pulmonary function, activities of daily living (ADL), in-hospital mortality rate and 6-month all-cause mortality rate.

**Discussion:**

This randomized, controlled, clinical trial will assess whether CR improves physical function and reduces rehospitalization in patients hospitalized in CICU with ADHF. The results will provide further research-based evidence for the clinical application of CR in patients with ADHF.

**Trial Registration:**

Chinese Clinical Trial Registry ChiCTR2100050151. Registered on 19 August 2021.

## Introduction

Heart failure (HF) remains a major public health issue worldwide. The prevalence of HF still continues to raise due to a growing and aging population and improvements in the treatment of cardiovascular diseases (1). Acute decompensated heart failure (ADHF), defined as the new onset or worsening of symptoms and signs of HF, is one of the leading causes of hospitalization (2, 3). Despite the drugs and device-based therapy have substantially improved over recent decades, the mortality and rehospitalization rates are still high in patients with ADHF (4–6).

Impaired exercise capacity in patients with ADHF has detrimental effects on the health-related quality of life (QOL) and adverse outcomes (7). In recent decades, the physiological basis of exercise benefits was acknowledged, which led to the development of cardiac rehabilitation (CR) (8). There is solid evidence from numerous trials and meta-analyses that exercise-based CR improves cardiorespiratory function, exercise tolerance and QOL in patients with chronic HF (9, 10). Several studies showed a reduced rehospitalization after the setting of CR in patients with chronic HF (11, 12). Exercise-based CR is strongly recommended (Class I, Level A) to improve exercise capacity and QOL as well as reduce HF hospitalization in patients with chronic HF by the 2021 European Society of Cardiology (ESC) guidelines for the diagnosis and treatment of acute and chronic HF (13). However, data on the efficacy and safety of CR in hospitalized patients with ADHF is limited.

Recently, the REHAB-HF trial showed tailored rehabilitation intervention improved the physical function in old patients hospitalized with ADHF (14, 15). In REHAB-HF trial, patients admitted to the intensive care unit were excluded. However, the management of ADHF usually requires intensive care. We hypothesize early CR will improve physical function and reduce mortality and rehospitalization in patients who enter the cardiac intensive care unit (CICU) with ADHF. The Short Physical Performance Battery (SPPB) is a well-established tool to evaluate physical performance and has predictive value in hospitalization, disability and mortality in patients with HF (16, 17). Therefore, SPPB score will be used as one of the primary endpoints of this protocol. In addition, this trial will evaluate whether CR intervention would improve 6-month rehospitalization, cardio-pulmonary function, activities of daily living (ADL), in-hospital mortality rate and 6-month all-cause mortality rate among patients with ADHF admitted to CICU.

## Materials and Methods

### Study Design

This is a single-center, randomized controlled, single-blind clinical trial, which is designed to evaluate the efficacy and safety of CR in patients hospitalized in CICU with ADHF. This study protocol follows the principles of the Standard Protocol Items: Recommendations for Intervention Trials (SPIRIT) statement (18). This study has been approved by the Ethics Committee of Xiamen Cardiovascular Hospital of Xiamen University (approved number: 2021YLK6) and registered at Chinese Clinical Trial Registry (registration number: ChiCTR2100050151).

**Figure 1:**
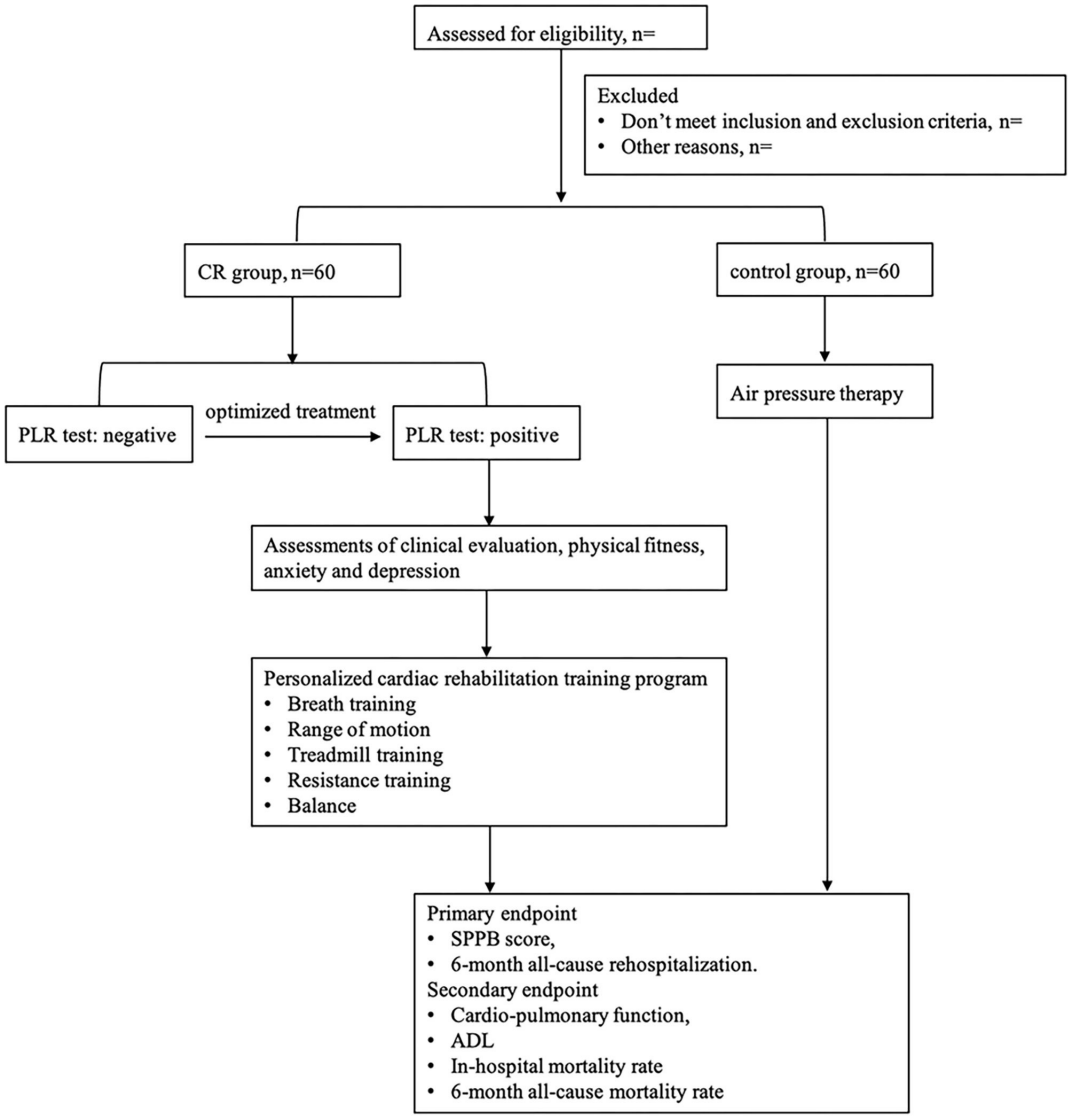
Study design. CR, cardiac rehabilitation; PLR, passive leg raise; SPPB, short physical performance battery; ADL, activities of daily living.

This study includes a 5-day intervention period and a 6-month follow-up period. A total of 120 patients hospitalized in CICU with ADHF will be randomly assigned to the two groups with a 1:1 ratio. Participants will receive tailored and progressive CR intervention or attention control. Clinical outcome evaluation will be conducted before and after intervention and at 6 months. The flow diagram of the study protocol is outlined in Figure 1.

### Participants

Participants will be recruited in the CICU of the Affiliated Cardiovascular Hospital of Xiamen University. Subjects hospitalized with ADHF will be screened according to the inclusion and exclusion criteria. The eligible subjects who agreed to participate in this study will be further determined by a cardiologist.

### Inclusion Criteria

(1) Age ≥ 18 years old;

(2) Acute decompensated heart failure was defined according to the “2021 ESC Guidelines for the diagnosis and treatment of acute and chronic heart failure” (13). Combined with symptoms, signs and laboratory results, and meet all the following conditions:

(2.1) At least one symptom was worsened from baseline: (a) dyspnea (dyspnea after exercising, paroxysmal nocturnal dyspnea or orthopnea), (b) venous congestion of systemic circulation (edema of lower extremities, liver congestion, ascites), and (c) tissue hypo-perfusion (oliguria or anuria, cold clammy limbs, consciousness disorder, hyper-lacticacidemia, or metabolic acidosis);

(2.2) At least one of the following signs of heart failure: (a) pulmonary edema on exam or by chest X ray, (b) Increased b-type natriuretic peptide or N-terminal prohormone BNP, and (c) abnormal cardiac structure and/or function by cardiac ultrasound;

(3) The symptoms and signs are consistent with acute decompensated heart failure judged by the investigator;

(4) Patients who have New York Heart Association Cardiac Function class III or IV symptoms at admission;

(5) Clinical stability has been achieved for 24 h after optimizing treatment;

(6) Patient was independent with basic activities of daily living including the ability to ambulate independently (with or without the use of an assistive device) prior to admission.

### Exclusion Criteria

(1) Acute coronary syndrome within 2 days; onset of chest pain within 8 h; or combined with mechanical complications;

(2) Fatal arrhythmia, high-grade atrioventricular block, new onset atrial fibrillation or atrial flutter;

(3) Severe aortic valve stenosis;

(4) Acute myocarditis, acute pericarditis, or acute infective endocarditis;

(5) Severe hypertrophic obstructive cardiomyopathy;

(6) Patients with chronic kidney disease stage 4 and 5 undergoing long-term hemodialysis or requiring long-term hemodialysis within the next 6 months;

(7) A recent embolism, atrium, or ventricular thrombosis, thrombophlebitis;

(8) Patients who require a pacemaker, implantable cardioverter defibrillator (ICD), or ventricular assist device, or those awaiting cardiac transplantation within the next 6 months;

(9) Those who had received standardized cardiac rehabilitation within the past 6 months;

(10) Severe cognitive impairment (MMSE ≤ 9 points);

(11) Inability or unwillingness to comply with the study requirements;

(12) Patients who dropped out or discharged during the study period.

### Sample Size

According to the results of the previous study, the baseline SPPB sores was 6.0 ± 2.8 in CR group and 6.1 ± 2.6 in control group, the SPPB sores at 3 months was 8.3 ± 0.2 in CR group and 6.9 ± 0.2 in control group (14). To minimize both false positive and false negative errors, the type-I error rate (α) is set at 0.01 instead of 0.05 and the type-II error rate is set at 0.1 instead of 0.2. The sample size is calculated according to the following formula: *n* = 2σ^2^ × *f* (α,β)/(μ_1_-μ_2_)^2^ (19). The sample size of each group was estimated to be 44 per group. Taking approximate a 20% drop-off rate and a 7% missing rate into account, the final sample size was set to 60 per group. Therefore, a total of 120 subjects will be recruited in this study.

### Randomization, Allocation Concealment, and Blinding

A random allocation sequence will be generated by an independent, blinded statistician using research randomizer (http://www.randomizer.org). Sequentially numbered, opaque, sealed envelopes (SNOSE) are used to achieve allocation concealment. One research member will independently allocate the participants to groups in a 1:1 ratio by opening the envelopes sequentially. The primary and secondary outcomes will be assessed by researchers blinded to the group allocations.

### Intervention

Clinical evaluation will be conducted before each rehabilitation training session. Those who pass the following evaluation will enter the rehabilitation program: no onset or recurring chest pain in the past 8 h; no onset symptoms of decompensated heart failure; new onset arrhythmia or dynamic changes of electrocardiogram (ECG) in the past 8 h; no further elevation in troponin levels; resting heart rate <110 beats per minute, resting blood pressure (BP) 90–150/60–100 mmHg and blood oxygen saturation (SaO_2_) ≥95%. Then, physical fitness assessments will be performed before rehabilitation program which include lung function, grip strength, maximum strength, flexibility, coordination, and balance. Additionally, assessment of anxiety and depression using Patient Health Questionnaire−9 (PHQ-9) and Generalized Anxiety Disorder−7 Questionnaire (GAD-7) will be evaluated before rehabilitation program.

In control group, subjects without contraindications will receive air pressure therapy to prevent deep vein thrombosis. The impedance cardiography (ICG) is a reliable, repeatable and non-invasive method to monitor cardiodynamic data including stroke volume (SV), end diastolic filling rate (EDFR) and peripheral vascular resistance (SVR) (20, 21). Passive Leg Raising (PLR) test, a reversible fluid-loading procedure to test fluid responsiveness, is used to ensure the safety of phase I cardiac rehabilitation (22, 23). Patients randomized to the CR group will start the exercise program if the PLR test is positive. Optimized treatment will be taken in patients with negative result of the PLR test. CR program will be started once the PLR test turn to positive in this part of patients. Personalized CR training will be performed based on the state of mind, New York Heart Association (NYHA) classification and physical fitness assessments. Whether the subjects will enter into the next level training program is according to the heart rate response, Borg scale score and Perme score (Table 1). A rehabilitation therapist will closely monitor the ECG, BP, SaO_2_, and ICG parameters (SV, EDFR, and SVR) at the bedside during the rehabilitation exercise training. Rehabilitation training will be stopped if the following situations occur: new onset arrhythmia or heart rate rise ≥20 beats per minutes or dynamic changes of ECG; systolic BP drops or rises more than 40 mmHg; SaO_2_ < 95%; a continuous downward trend in SV more than 1 min; onset symptoms of exercise intolerance such as chest tightness, heart palpitations, and dyspnea.

**Table 1:** Personalized cardiac rehabilitation program.

	**Level**	**Level 1**	**Level 2**	**Level 3**	**Level 4**	**Level 5**	**Level 6**	**Level 7**
Assessment	Unconscious	Unconscious	Conscious	Conscious	Conscious	Conscious	Conscious	Conscious
	Muscle strength	Upper limb muscle strength <3 level	Upper limb muscle strength ≥3 level	Limb muscle strength ≥ 3 level	Limb muscle strength ≥4 level	Limb muscle strength ≥4 level	Lower limbs muscle strength 5 level	Lower limbs muscle strength 5 level
	NYHA classification	III–IV	III–IV	III	II–III	II–III	II–III	II–III
Rehabilitation plans and programs	Breath training	/	/	Progressive breathing muscle training	Progressive breathing muscle training	Progressive breathing muscle training	Progressive breathing muscle training	Progressive breathing muscle training
	Range of Motion (ROM)	Passive rom once per day	Active/passive rom once per day	Active rom once per day	Active rom once per day	Active rom once per day	Active rom once per day	Active rom once per day
	Treadmill training	Passive bed treadmill training for 10–20 min	Passive bed treadmill training for 10–20 min	Bed treadmill training for 10–20 min	Bedside treadmill training for 10–20 min	bedside treadmill training for 10–20 min	Bedside treadmill training for 10–20 min	Bedside treadmill training for 10–20 min
	Resistance training	/	/	Progressive resistance training 15 times per group (elbow flexion and extension movements), 3 groups, rest for 2 min between each group	Progressive resistance training 15 times per group (elbow flexion and extension movements for 3 groups and abdominal crunch for 1 group and hip bridge for 1 group), rest for 2 min between each group	Progressive resistance training 15 times per group (elbow flexion and extension movements for 3 groups and abdominal crunch for 1 group and hip bridge for 1 group), rest for 2 min between each group	Progressive resistance training 15 times per group (elbow flexion and extension movements for 3 groups and abdominal crunch for 1 group and hip bridge for 1 group), rest for 2 min between each group	Progressive resistance training 15 times per group (elbow flexion and extension movements for 3 groups and abdominal crunch for 1 group and hip bridge for 1 group), rest for 2 min between each group
	Sitting in bed (bed head elevation >45°)	/	Sitting in bed 5 min, 2 times per day	Sitting in bed 5 min, 2 times per day	Sitting in bed 5 min, 2 times per day	Sitting in bed 5 min, 2 times per day	Sitting in bed 5 min, 2 times per day	Sitting in bed 5 min, 2 times per day
	Sitting at the edge of the bed	/	/	Sitting at the edge of the bed 5 min, 2 times per day	Sitting at the edge of the bed 5 min, 2 times per day	Sitting at the edge of the bed 5 min, 2 times per day	Sitting at the edge of the bed 5 min, 2 times per day	Sitting at the edge of the bed 5 min, 2 times per day
	Standing and stepping	/	/	/	Standing/stepping for 2 min	Standing/stepping for 2 min	Standing/stepping for 5 min	Standing/stepping for 5 min
	Bed to chair transfer	/	/	/	Sitting in chair 5 min per day, 2 times per day	Sitting in chair 5 min per day, 2 times per day	Sitting in chair 5 min per day, 2 times per day	Sitting in chair 5 min per day, 2 times per day
	Walking bedside bed	/	/	/	/	Walking training by supported by walker for 2 min	Walking training beside bed for 2 min	Walking training for 2 min
Goal	Heart rate response (compared with resting heart rate)	Increase 5–15 beats per minute	Increase 5–15 beats per minute	Increase 20–30 beats per minute	Increase 20–30 beats per minute	Increase 20–30 beats per minute	Increase 20–30 beats per minute	Increase 20–30 beats per minute
	Borg scale score		<12	12–13	12–13	12–13	12–13	12–13
	Perme score	/	Sitting up on bed for a score of 3	Meditation balance for a score of 3	Sit-to-stand transfer or standing balance for a score of 3	Bed to chair transfer for a score of 3	Walking training for a score of 3	/

### Outcomes

The outcomes will be assessed by independent researchers who have been trained before the study. The primary outcomes are the SPPB score and 6-month all-cause rehospitalization. The secondary outcomes include cardio-pulmonary function, activities of daily living (ADL), in-hospital mortality rate and 6-month all-cause mortality rate. Cardiac function indicators are as follow: 6-min walking test (6 MWT) before discharge, SV before discharge, EDFR, SVR, NYHA classification, biochemical markers of myocardial necrosis, N-terminal brain natriuretic peptide (NT-pro BNP), and left ventricular ejection fraction (LVEF). Pulmonary function indicators include 1 second rate of transfer/discharge (FEV1/FVC) and maximum ventilation volume per minute (MVV). In addition, we will evaluate the Short-Form 36-Item Health Survey (SF-36), psychological assessment, nutritional assessment and cardio-cerebrovascular events (Table 2).

**Table 2:** The follow-up schedule.

	**Baseline**	**1 month**	**3 months**	**6 months**
Smoking	X			
Sports	X			
Participate in phase II cardiac rehabilitation				
Psychological assessment	X		X	X
Nutritional assessment	X	X		X
Rehospitalization				
Cardio-cerebrovascular events				
36-item short-form (SF-36)	X	X	X	

### Data Management

An EpiData database will be established based on the CRFs project. The researchers will fill in relevant information back-to-back timely and accurately. The study data will be monitored by researchers at the School of Public Health of Xiamen University every 3 months.

### Data Analysis

Statistical analysis will be conducted using SPSS 25.0 (IBM, Chicago, IL, USA) statistical software. *P* < 0.05 (two-sided) was considered to be statistically significant. Normally distributed continuous variables were presented as mean ± SD and evaluated with paired or unpaired Student's *t* test. Non-normally distributed continuous variables were expressed as median with interquartile range (IQR) and determined by Wilcoxon signed-rank test or Mann-Whitney U test. Categorical variables were recorded as frequency and percentage (*N*, %) and performed by χ^2^ test or Fisher's exact test as appropriate.

## Discussion

This is a study protocol for a single-center, randomized controlled, single-blind clinical trial to evaluate the efficacy and safety of phase I CR in patients hospitalized in CICU with ADHF. The results of this study will provide reliable evidence for tailored and progressive CR intervention in patients with ADHF.

CR is one of the treatment strategies for HF, which services as one way to reduce the financial burden of HF treatment (24). A recent trial showed CR could improve physical function in patients with ADHF, especially those in NYHA Class II group (14). CICU is usually needed for the patients with ADHF presented with signs of respiratory distress, hypoperfusion, and hemodynamic instability. These patients are at high risk for unexpected in-hospital mortality and re-hospitalization (25). Whether personalized, tailored, and progressive CR intervention is associated with improved QOL and outcomes in patients in CICU with ADHF has not yet been clarified. Thus, evidence on the efficacy and safety of CR intervention is needed in individuals enter the CICU with ADHF.

Tailoring exercise prescription based on the characteristics of patients is necessary for the development of CR in ADHF. Improved SPPB score was related to better prognosis in patients with HF (26). The PEARL study and the REHAB-HF trial showed CR could improve the SPPB score among elderly patients with ADHF (14, 27). However, re-hospitalization rate was not included as a study endpoint in PEARL study. In REHAB-HF trial, reduced re-hospitalization rate was not observed in CR group during the follow-up period. In addition, the majority of CR interventions were conducted in the outpatient setting in REHAB-HF trial. We hypothesize early phase I CR training will increase the SPPB score and reduce the re-hospitalization rate. Additionally, our personalized, tailored and progressive CR program include comprehensive physical fitness interventions. The physical fitness interventions include strength, flexibility, coordination, and balance training. The psychological interventions will be performed by specialized psychological intervention therapists. To achieve the outcomes, strict follow-up procedure will be conducted for 6 months. These measures could promote individualized CR intervention in patients with ADHF.

In conclusion, we aim to explore the efficacy and safety of CR in patients with ADHF hospitalized in CICU through this clinical trial. The research results are expected to help provide further evidence for clinical application and guidelines. Subsequent studies will need to demonstrate sufficient efficacy and safety of CR in patients with ADHF.

## Ethics Statement

The studies involving human participants were reviewed and approved by the Ethics Committee of Xiamen Cardiovascular Hospital of Xiamen University. The patients/participants provided their written informed consent to participate in this study.

## Author Contributions

LW and CD have conceived and designed the study. LW, JL, LC, MX, and YZ will recruit and screen the participants. LW, JL, LC, FM, and HJ will participate in the data collection. JL and LC will participate in the data analysis. LW and PZ participated in drafting this manuscript. ZS, PZ, and CD provided the supervision support. All authors contributed to the critical revisions and final approval of the manuscript.

## Funding

This study was supported by the Xiamen Medical and Health Guidance Project (3502Z20209146), Xiamen Medical and Health Key Project (3502Z20204005), and Fujian Provincial Science and Technology Project (2018D0013). The funders had no role in study design, data collection and analysis, decision to publish and preparation of the manuscript.

## Author Disclaimer

The content is solely the responsibility of the authors and does not necessarily reflect the views of the organizations listed above.

## Conflict of Interest

The authors declare that the research was conducted in the absence of any commercial or financial relationships that could be construed as a potential conflict of interest.

## Publisher's Note

All claims expressed in this article are solely those of the authors and do not necessarily represent those of their affiliated organizations, or those of the publisher, the editors and the reviewers. Any product that may be evaluated in this article, or claim that may be made by its manufacturer, is not guaranteed or endorsed by the publisher.
